# GABA Regulation of Burst Firing in Hippocampal Astrocyte Neural Circuit: A Biophysical Model

**DOI:** 10.3389/fncel.2019.00335

**Published:** 2019-07-23

**Authors:** Junxiu Liu, Liam McDaid, Alfonso Araque, John Wade, Jim Harkin, Shvan Karim, David C. Henshall, Niamh M. C. Connolly, Anju P. Johnson, Andy M. Tyrrell, Jon Timmis, Alan G. Millard, James Hilder, David M. Halliday

**Affiliations:** ^1^School of Computing, Engineering and Intelligent Systems, Ulster University, Derry, United Kingdom; ^2^Department of Neuroscience, University of Minnesota, Minneapolis, MN, United States; ^3^Department of Physiology and Medical Physics, Royal College of Surgeons in Ireland, Dublin, Ireland; ^4^FutureNeuro Research Centre, Royal College of Surgeons in Ireland, Dublin, Ireland; ^5^Department of Electronic Engineering, University of York, York, United Kingdom

**Keywords:** astrocyte cell, GABA interneuron, burst firing, calcium oscillation, potentiation

## Abstract

It is now widely accepted that glia cells and gamma-aminobutyric acidergic (GABA) interneurons dynamically regulate synaptic transmission and neuronal activity in time and space. This paper presents a biophysical model that captures the interaction between an astrocyte cell, a GABA interneuron and pre/postsynaptic neurons. Specifically, GABA released from a GABA interneuron triggers in astrocytes the release of calcium (*Ca*^2+^) from the endoplasmic reticulum via the inositol 1, 4, 5-trisphosphate (*IP*_3_) pathway. This results in gliotransmission which elevates the presynaptic transmission probability rate (PR) causing weight potentiation and a gradual increase in postsynaptic neuronal firing, that eventually stabilizes. However, by capturing the complex interactions between *IP*_3_, generated from both GABA and the 2-arachidonyl glycerol (2-AG) pathway, and PR, this paper shows that this interaction not only gives rise to an initial weight potentiation phase but also this phase is followed by postsynaptic bursting behavior. Moreover, the model will show that there is a presynaptic frequency range over which burst firing can occur. The proposed model offers a novel cellular level mechanism that may underpin both seizure-like activity and neuronal synchrony across different brain regions.

## 1. Introduction

Spiking neural networks (SNNs) are considered to be the most biologically plausible representation of brain function (Ghosh-dastidar and Adeli, 2009). Additionally, SNNs capture a Hebbian type learning paradigm where the timing between pre- and post-synaptic spikes dictates whether synaptic depression or potentiation occurs (Song et al., 2000). SNNs have also been shown to be effective in time series prediction (Reid et al., 2014), spatiotemporal pattern recognition (Hu et al., 2013), and system control (Liu et al., 2015) in various application domains. In SNNs, the neurons and synapses are fundamental components in the network, where the information is encoded in spikes or action potentials for transmission between neurons (Izhikevich, 2003). In the central nervous system neurons receive input stimuli and respond by firing spike patterns such as bursting, which has been observed in the hippocampus of rodents (Miles and Wong, 1986), electric fish (Gabbiani et al., 1996), and in the primary motor cortex, brainstem and thalamus within the somatomotor system of humans (Arichi et al., 2017). The bursts can, in some cases, represent normal brain function and in other cases abnormal brain function (e.g., epilepsy) (Araque et al., 1999; Halassa et al., 2007).

Research has shown that astrocytes, one type of glial cell, modulate neuronal activity (Halassa et al., 2007; Breslin et al., 2018; Flanagan et al., 2018) where a single astrocyte may enwrap a large number of synapses (~10^5^ synapses), and connect to several neighboring neurons (four-eight). The interplay between an astrocyte and the neighboring neurons is believed to occur at the tripartite synapse (Araque et al., 1999), which is bi-directional and serves, in some cases, to modulate the synaptic transmission probability rate (PR): via the direct/indirect retrograde signaling messenger endocannabinoids (Wade et al., 2012). This gives rise to re-modeling of the SNN connectivity (Wade et al., 2011; Naeem et al., 2015; Johnson et al., 2018; Liu et al., 2018).

It has also been reported that gamma-aminobutyric acidergic (GABA) interneurons participate in astrocyte-mediated control of excitatory synaptic transmission (Perea et al., 2016) and exercises control over the firing frequency of pyramidal cells. Furthermore GABA release synchronizes principal cell population discharge contributing to the generation of rhythmic activity in neuronal networks, such as theta and gamma frequency oscillations (Kullmann, 2011). A recent paper reported that GABA released in proximity to a tripartite synapse can activate GABA-B receptors on the astrocyte leading to gliotransmission, which is known to regulate synaptic transmission probability (Liu et al., 2018). The research reported in Kurosinski and Götz (2002), Kullmann (2011), Liu et al. (2018) provides the underpinning for the work presented here.

In this paper, we investigate the coupling between a GABA interneuron, an astrocyte terminal and the pre and postsynaptic terminals. The main contributions of this paper include (i) a novel biophysical model that describes the signaling pathways at the tripartite synapse and (ii) a novel mechanism that can potentially explain postsynaptic neuron burst firing. The rest of paper is organized as follows. Section 2 presents the biophysical model while section 3 provides simulation results that demonstrate the bursting. Section 4 concludes the paper and discusses future work.

## 2. Biophysical Model of a Network Bursting

In this section, a detailed discussion of the signaling pathways at the tripartite synapse is presented with a specific focus on GABA signaling between the presynaptic terminal and the nearby astrocyte. It will be shown that this interplay acts as a frequency dependent switch, which modulates the probability of release (PR) at the presynaptic terminal. Our *Ca*^2+^ dynamics model shows that calcium (*Ca*^2+^) oscillations only occur over a range of inositol 1, 4, 5-trisphosphate (*IP*_3_) concentrations and furthermore this paper will show that *Ca*^2+^ oscillations are periodic and this behavior is key to the bursting behavior.

### 2.1. Signaling Pathways and Activity Regulations

**Figure 1:**
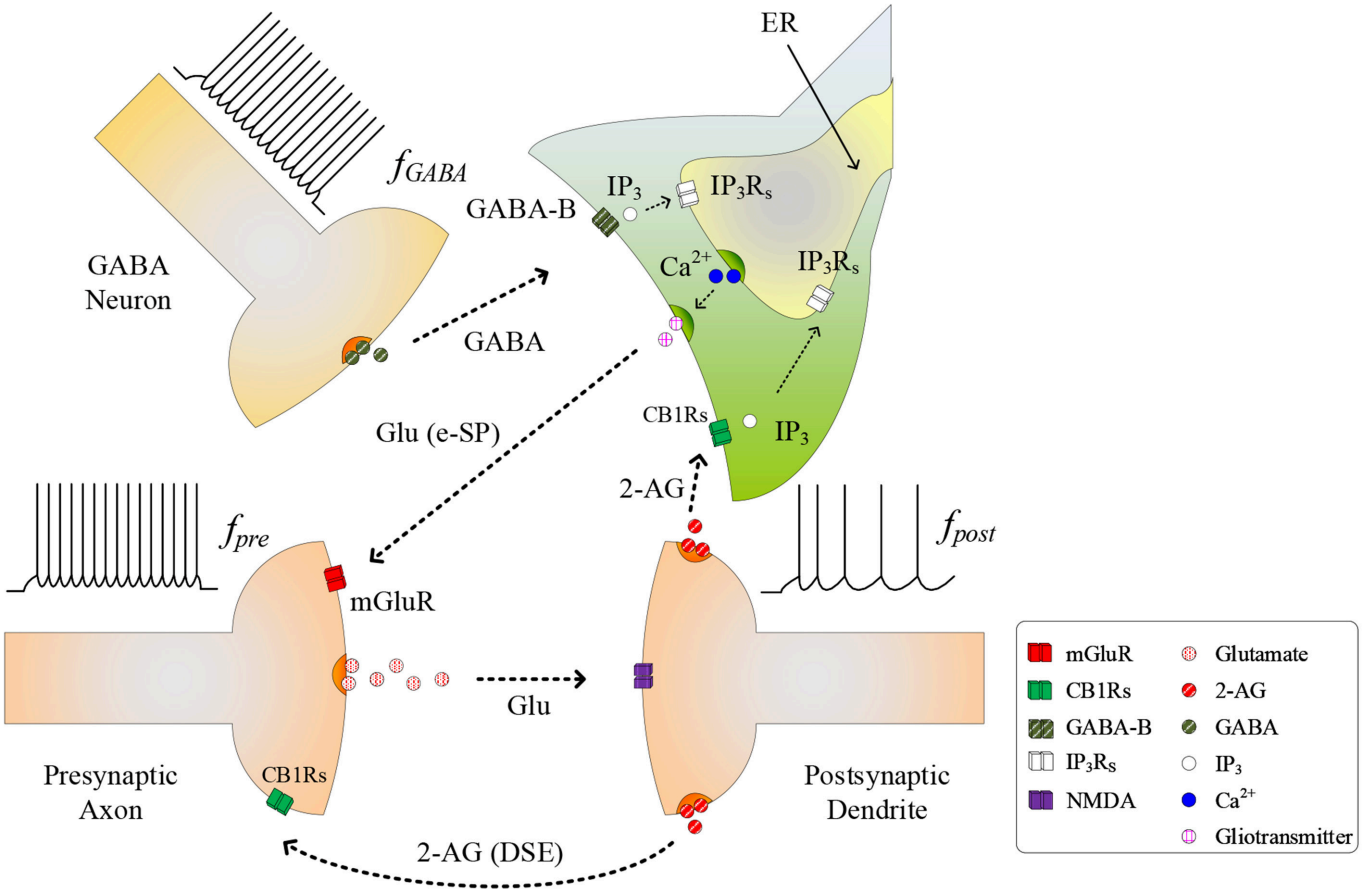
Signaling pathways at the tripartite synapse where GABA released from GABA interneuron binds to GABA-B receptors on the astrocyte membrane and $IP_{3}^{GABA}$ is released into the astrocyte cytosol. 2-AG released from the postsynaptic neuron binds to the receptors on the astrocyte membrane triggering the generation of $IP_{3}^{AG}$. With *f*_*post*_ low and *IP*_3_ reached a level, *IP*_3_ induces *Ca*^2+^ release from the ER and gliotransmitter is released into the synaptic cleft [Glu (e-SP) pathway] where it binds to mGluR receptors on the presynaptic membrane. This causes an increase in PR and the plasticity window opens.

The conventional tripartite synapse has three terminals: the presynaptic axon, postsynaptic dendrite and the astrocyte cell (Wade et al., 2012; Liu et al., 2018). In this paper we consider earlier work where, in a hippocampal astrocyte neural network, GABA interneurons interact with excitatory tripartite synapses to dynamically change the synaptic transmission behavior from inhibitory to excitatory through modulation of PR (Perea et al., 2016). The signaling pathways between the GABA interneuron and tripartite synapse are shown in Figure 1. When an input stimulus of frequency (*f*_*pre*_) is present at the excitatory presynaptic axon, neurotransmitter (glutamate) is released into the cleft and subsequently binds to receptors at the postsynaptic dendrite causing the depolarization of the postsynaptic neuron. While the authors accept that fast-spiking interneurons can fire at much higher frequencies than glutamatergic neurons, in this work we assume for simplicity that the firing rate of the GABA interneuron (*f*_*GABA*_) follows *f*_*pre*_, as the most likely physiological condition would be the activation of GABA interneuron by activation of glutamatergic axons (Serrano et al., 2006; Covelo and Araque, 2018). While GABA initially binds to GABA-A receptors inhibiting synaptic transmission and post-synaptic neuronal activity, recent work (Perea et al., 2016) has shown that with repeated firing of GABA interneurons, GABA also binds to GABA-B receptors on the astrocyte membrane, resulting in a switch from inhibition to excitation at the presynaptic terminal, and an associated excitatory response at the postsynaptic terminal. In this paper we focus on astrocyte-mediated GABA-induced excitation since the postsynaptic inhibition was found negligible, and the transient acute presynaptic inhibition was overpowered by the astrocyte signaling during sustained activity (Perea et al., 2016). Hence, our model dos not incorporate these negligible or transient inhibitory effects, focusing in the sustained mechanisms and effects of inhibitory signaling through astrocyte activation.

As *f*_*GABA*_ increases, the GABA concentration level in the extracellular space increases, and a level is reached whereby binding to GABA-B receptors on the astrocyte membrane commences, leading to the production of *IP*_3_: we subsequently refer to *IP*_3_ due to GABA as $IP_{3}^{GABA}$, which contributes to the overall cytosolic *IP*_3_ (Perea et al., 2016). $IP_{3}^{GABA}$ is a secondary messenger which is degraded when released into the cytoplasm: initially cytosolic *Ca*^2+^ and *IP*_3_ levels are low and therefore degradation of *IP*_3_ will also be low, as the degradation rate correlates with both *Ca*^2+^ and *IP*_3_ concentrations. This degradation is gradually overcome with increasing levels of GABA and the *PLCδ* signaling pathway, which is modulated by *Ca*^2+^ and is accounted for in this work. Finally $IP_{3}^{GABA}$ starts to bind to *IP*_3_ receptors (*IP*_3_*R*_*s*_) on the Endoplasmic Reticulum (ER). When the total cytosolic *IP*_3_ is sufficiently high, *Ca*^2+^ is released from the ER (De Pittà et al., 2009). At some point both *IP*_3_ and *Ca*^2+^ reaches a level at which an oscillating Calcium-Induced Calcium Release (CICR) occurs from the ER (Marchant et al., 1999): hereafter referred to as *T*_*CICR*_. Several mechanisms are believed to contribute to *Ca*^2+^ oscillation but there is still much debate around this topic. For example, *IP*_3_*R*_*s*_ have binding sites for both *IP*_3_ and *Ca*^2+^, and *Ca*^2+^ release from the ER is believed to rely on coincidence binding of these ions. The time between *IP*_3_ and *Ca*^2+^ binding depends on the concentration of these ions and therefore this could explain why *Ca*^2+^ is believed to be a regulator of *IP*_3_*R*_*s*_ activity: at low *Ca*^2+^ levels *IP*_3_*R*_*s*_ activity is increased, whereas the opposite is true at high *Ca*^2+^ levels. This is in agreement with other research (Dawson, 1997). However, there is not enough experimental evidence on these receptors to formulate a sufficiently detailed model. Therefore, in this work we revert to a hitherto accepted model (Perea et al., 2016) where *Ca*^2+^ oscillatory behavior is believed to arise from the feedback interplay between *Ca*^2+^, *IP*_3_, and *IP*_3_ degradation. As *Ca*^2+^ and *IP*_3_ rapidly increase there is a complex dependency between the concentrations of both *Ca*^2+^ and *IP*_3_ and *Ca*^2+^/*IP*_3_-induced degradation of *IP*_3_, which is the dominant process at elevated *Ca*^2+^/*IP*_3_ levels. Therefore, a transient elevation of *Ca*^2+^ and/or *IP*_3_ is followed by a rapid drop in *IP*_3_, which can reduce *IP*_3_ to below *T*_*CICR*_. At this point degradation of *IP*_3_ is weak because both the *Ca*^2+^ and *IP*_3_ levels have fallen and therefore *IP*_3_ starts to increase again due to $IP_{3}^{GABA}$. When the *T*_*CICR*_ level is reached again a transient elevation of *Ca*^2+^ re-occurs. We will demonstrate that our results support this behavior. This oscillatory behavior causes the release of the glutamate from the astrocyte (gliotransmitter) into the synapse [see Glu (e-SP) pathway in Figure 1], which binds to pre-synaptic group I metabotropic Glutamate Receptors (mGluRs) at the presynaptic terminal. This signaling pathway results in an increase in PR at the presynaptic terminal (Navarrete and Araque, 2010).

As PR increases more glutamate is released into the cleft and potentiation/depression of the synaptic weight can commence with the availability of glutamate to bind to N-methyl-D-aspartate (NMDA)-type glutamate receptors (Lüscher and Malenka, 2012). While we acknowledge that the biophysical mechanisms regulating the functional dependency between PR and plasticity are complex and not fully understood, we propose that PR acts as a “switch” which can turn on/off potentiation/depression at synaptic sites. To formulate a tractable mathematical model that captures this relationship we modulate the height of the Spike Timing Dependent Plasticity (STDP) associated plasticity window using PR: with *PR*≥PR^*^ (PR^*^ is defined as the plasticity activation level) the plasticity window fully opens and with *PR*<PR^*^ the plasticity window closes. The decision on whether potentiation or depression occurs is governed by the STDP rule (Magee and Johnston, 1997) where potentiation occurs when the presynaptic spike precedes postsynaptic spike, otherwise depression occurs. Additionally, we consider the case where the postsynaptic neuron is sufficiently depolarized such that the retrograde messenger 2-arachidonyl glycerol (2-AG) is released from the postsynaptic neuron. Since the contribution of 2-AG signaling to the observed GABA-mediated regulatory effects of astrocytes on excitatory transmission is negligible (Perea et al., 2016), the authors take the view that 2-AG signaling onto GABAergic terminals would not be a significant factor in network bursting. However, we do consider 2-AG binding to type 1 Cannabinoid Receptors (CB1Rs) on the astrocyte membrane which then initiates the release of the *IP*_3_ into the cytoplasm of the astrocyte: we denote this secondary messenger as $IP_{3}^{AG}$.

**Figure 2:**
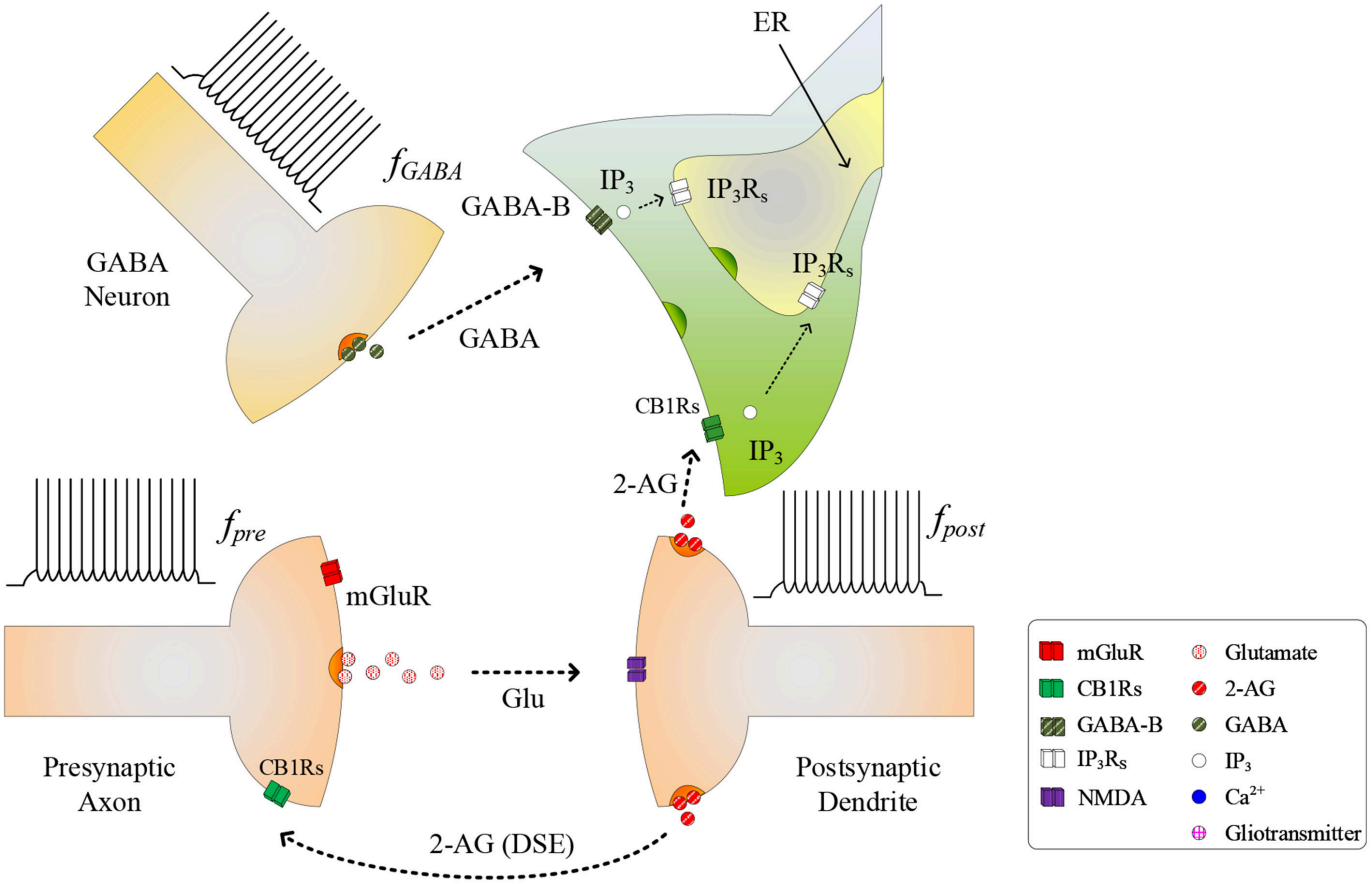
Signaling pathways of the tripartite synapse where for high *f*_*post*_ *T*_*CICR*_ is reached and hence the *Ca*^2+^ oscillation and the Glu (e-SP) pathway ceases causing PR to fall and the plasticity window shuts: PR also falls due to the increase in the 2-AG (DSE) pathway. Under this condition the level of neurotransmitter in the cleft falls to baseline and *f*_*post*_ diminishes.

During the synapse learning phase, the frequency of the postsynaptic neuron, *f*_*post*_, is increasing, as is the 2-AG signal and consequently $IP_{3}^{AG}$. As $IP_{3}^{AG}$ contributes to the total *IP*_3_, *IP*_3_ will eventually reach a level where degradation of *IP*_3_ no longer reduces *IP*_3_ (and therefore *Ca*^2+^) to below *T*_*CICR*_. In this instance, both the oscillatory *Ca*^2+^ transient and Glu (e-SP) pathways cease (Liu et al., 2018) (see Figure 2). In addition, the released 2-AG also binds to CB1Rs on the presynaptic terminal triggering the Suppression of Excitation (DSE) pathway, and results in a decrease in PR (Alger, 2002). Due to the reduction in the Glu (e-SP) pathway and the increase in the DSE pathway, PR decreases at the presynaptic terminal, the level of neurotransmitter in the cleft then falls to baseline and the frequency of the postsynaptic *f*_*post*_ diminishes which in turn causes $IP_{3}^{AG}$ to reduce. Furthermore, the total *IP*_3_ degrades due to cytosolic degradation pathways including *IP*_3_ 3-kinase $IP_{3}^{3K}$, and dephosphorylation by inositol polyphosphate 5-phosphatase ($IP_{3}^{5P}$) (see Equation 11). Together, these processes reduce *IP*_3_ levels below *T*_*CICR*_, and the rate of degradation diminishes sufficiently to allow *IP*_3_ to increase again due to $IP_{3}^{GABA}$. When the *T*_*CICR*_ level is again exceeded, *Ca*^2+^ oscillations re-commence, the Glu (e-SP) pathway is re-established, PR increases and the level of neurotransmitter in the cleft is raised. In this post-learning phase PR cannot be elevated to a level where the plasticity window opens (*PR*<PR^*^), as the postsynaptic neuron is active and therefore the 2-AG pathway leads to a reduction in PR due to the DSE pathway. Consequently, the postsynaptic neuron firing rate reaches a maximum when the *T*_*CICR*_ level is reached but it subsequently falls afterwards: a postsynaptic burst has occurred. This is followed by repeated bursts at each *Ca*^2+^ oscillatory period. We therefore propose that neuronal burst firing directly correlates with astrocytic *Ca*^2+^ oscillation.

Moreover, it should be noted that the duration of the burst correlated with the frequency of presynaptic terminal *f*_*pre*_ at the excitatory presynaptic axon. As *f*_*pre*_ increases so does the rate of increase of *IP*_3_ and the burst period is reduced. Therefore, network bursting is *f*_*pre*_ dependant and will only occur over a range of *f*_*pre*_.

### 2.2. Postsynaptic Neuron Model

In this paper, the Leaky Integrate and Fire (LIF) model (Gerstner and Kistler, 2002) is used due to the relatively low computing requirement and minimal parameters tuning. The LIF model is given by

(1) $$\begin{array}{r} \tau_{m}\frac{dv}{dt}=-v\left(t\right)+R_{m}\overset{n}{\underset{i=1}{\sum }}I_{syn}^{i}\left(t\right), \end{array}$$

where τ_*m*_ is the neuron membrane time constant, *v* is the neuron membrane potential, *R*_*m*_ is the membrane resistance, $I_{syn}^{i}$ is the current injected to the neuron membrane by *i*th synapse, and *n* is the total number of synapses associated with the neuron. When the neuron membrane potential *v* is greater than the firing threshold value, *v*_*th*_, the neuron fires and outputs a spike followed by a reset state or a refractory period (~2*ms*). The release of 2-AG correlates with the postsynaptic neuron activity (Naeem et al., 2015) and this is expressed as

(2) $$\begin{array}{r} \frac{d\left(AG\right)}{dt}=\frac{-AG}{\tau_{AG}}+r_{AG}\delta \left(t-t_{sp}\right), \end{array}$$

where *AG* denotes the released amount of 2-AG, τ_*AG*_, and *r*_*AG*_ are the 2-AG decay and production rates, and *t*_*sp*_ is the postsynaptic spike time. The released 2-AG binds to the CB1Rs at the presynaptic terminal and at the astrocyte terminal, and this will be discussed in section 2.4.

### 2.3. GABA Interneuron

The spike train at the presynaptic axon also presents at the GABA interneuron causing the release of GABA neurotransmitter (Perea et al., 2016), which can be described by

(3) $$\begin{array}{r} \frac{d\left(GABA\right)}{dt}=\frac{-GABA}{\tau_{GABA}}+r_{GABA}\delta \left(t-t_{sp}\right), \end{array}$$

where GABA denotes the released amount of the neurotransmitter GABA, τ_*GABA*_, and *r*_*GABA*_ are the GABA decay and production rates, and *t*_*sp*_ is the presynaptic spike arrival time. GABA binds to the GABA-B receptors at the astrocyte cell and this is modeled in the next subsection.

### 2.4. Astrocyte Cell

When GABA binds to GABA-B receptors on the astrocyte membrane, the amount of *IP*_3_ released is given by

(4) $$\begin{array}{r} \frac{d\left(IP_{3}^{GABA}\right)}{dt}=\frac{IP_{3}^{GABA*}-IP_{3}^{GABA}}{\tau_{ip3}^{GABA}}+r_{ip3}^{GABA}GABA, \end{array}$$

where $IP_{3}^{GABA}$ is the quantity of *IP*_3_ generated by GABA within the cytoplasm, $IP_{3}^{GABA*}$ is the baseline GABA level, $\tau_{ip3}^{GABA}$ is the decay rate of $IP_{3}^{GABA}$ and $r_{ip3}^{GABA}$ is the production rate of $IP_{3}^{GABA}$. When the postsynaptic neuron fires, the released 2-AG can also trigger *IP*_3_ generation (Wade et al., 2012) and this is modeled by

(5) $$\begin{array}{r} \frac{d\left(IP_{3}^{AG}\right)}{dt}=\frac{IP_{3}^{AG*}-IP_{3}^{AG}}{\tau_{ip3}^{AG}}+r_{ip3}^{AG}AG, \end{array}$$

where $IP_{3}^{AG}$ is the quantity of *IP*_3_ generated by 2-AG within the cytoplasm, $IP_{3}^{AG*}$ is the baseline level, $\tau_{ip3}^{AG}$ is decay rate of $IP_{3}^{AG}$, and $r_{ip3}^{AG}$ is production rate of $IP_{3}^{AG}$.

In addition, the *IP*_3_ production is also increased by the hydrolysis of the highly phosphorylated membrane lipid phosphatidylinositol 4, 5–bisphosphate (*PIP*_2_), such as the phosphoinositide-specific phospholipase C (PLC) isoenzyme of *PLCδ* (De Pittà et al., 2009). The *PLCδ* signaling is agonist independent and modulated by *Ca*^2+^ (De Pittà et al., 2009), and its activation rate can be modeled by

(6) $$\begin{array}{r} PLC\delta =PLC\delta^{\prime }Hill\left(Ca^{2+},K_{PLC\delta },2\right), \end{array}$$

where the maximum *PLCδ*-dependant *IP*_3_ production rate (De Pittà et al., 2009) can be modeled by

(7) $$\begin{array}{r} PLC\delta^{\prime }=\bar{PLC\delta^{\prime }}/\left(1+IP_{3}/K_{\delta }\right), \end{array}$$

and *K*_δ_ is the inhibition constant of *PLCδ* activity. The Hill function (De Pittà et al., 2009) is described by

(8) $$\begin{array}{r} Hill\left(x,K,n\right)\equiv \frac{x^{n}}{x^{n}+K^{n}}, \end{array}$$

where *n* is the Hill coefficient and *K* is the midpoint of the Hill function, namely the value of *x* at which *Hill*(*x, K, n*)|_*x* = *K*_ = 1/2.

The degradation of *IP*_3_ mainly occurs through phosphorylation into inositol 1, 3, 4, 5-tetrakisphosphate (*IP*_4_), catalyzed by *IP*_3_ 3-kinase (3K), and dephosphorylation by inositol polyphosphate 5-phosphatase (5P). The rate of *IP*_3_ degradation by $IP_{3}^{5P}$ (De Pittà et al., 2009) can be modeled by

(9) $$\begin{array}{r} IP_{3}^{5P}\approx {\bar{r}}_{5P}IP_{3}, \end{array}$$

where ${\bar{r}}_{5P}$ is the *IP*_3_ degradation rate by IP-5P. The activity of $IP_{3}^{3K}$ is regulated by *Ca*^2+^ in a complex fashion (De Pittà et al., 2009). The rate of *IP*_3_ degradation by $IP_{3}^{3K}$ can be modeled by

(10) $$\begin{array}{r} IP_{3}^{3K}={\bar{v}}_{3K}Hill\left(Ca^{2+},K_{D},4\right)Hill\left(IP_{3},K_{3},1\right), \end{array}$$

where ${\bar{v}}_{3K}$ is the maximum degradation rate by $IP_{3}^{3K}$, *K*_*D*_ is the *Ca*^2+^ affinity of $IP_{3}^{3K}$, and *K*_3_ is the *IP*_3_ affinity of $IP_{3}^{3K}$. Based on the previous contributions of *IP*_3_, the total *IP*_3_ is given by

(11) $$\begin{array}{r} IP_{3}=IP_{3}^{GABA}+IP_{3}^{AG}+PLC\delta -IP_{3}^{5P}-IP_{3}^{3K}. \end{array}$$

The Li-Rinzel model (Li and Rinzel, 1994) is used to model the *Ca*^2+^ dynamics within the astrocyte cell. The model consists of three channels, *J*_*chan*_, *J*_*leak*_, and *J*_*pump*_, where *J*_*chan*_ models the *Ca*^2+^ channel opening based on the mutual gating of the *Ca*^2+^ and *IP*_3_, *J*_*leak*_ models the *Ca*^2+^ leakage from the ER into the cytoplasm and *J*_*pump*_ models how *Ca*^2+^ is pumped out from the cytoplasm into the ER via Sarco-Endoplasmic-Reticulum *Ca*^2+^-ATPase (SERCA) pumps. The *Ca*^2+^ model in the approach of De Pittà et al. (2009) is used in this work, and it is described by

(12) $$\begin{array}{rcl} \frac{d\left(Ca^{2+}\right)}{dt} & = & J_{chan}\left(Ca^{2+},h,IP_{3}\right)+J_{leak}\left(Ca^{2+}\right)-J_{pump}\left(Ca^{2+}\right), \end{array}$$

(13) $$\begin{array}{rcl} \frac{dh}{dt} & = & \frac{h_{\infty }-h}{\tau_{h}}, \end{array}$$

where *J*_*chan*_ is *Ca*^2+^ release depending on the *Ca*^2+^ and *IP*_3_ concentrations, *J*_*pump*_ is the amount of stored *Ca*^2+^ within the ER via the SERCA pumps, *J*_*leak*_ is the *Ca*^2+^ leaking out of the ER and *h* is the fraction of activated *IP*_3_*R*_*s*_. The parameters *h*_∞_ and τ_*h*_ are given by

(14) $$\begin{array}{rcl} h_{\infty } & = & \frac{Q_{2}}{Q_{2}+Ca^{2+}}, \end{array}$$

(15) $$\begin{array}{rcl} \tau_{h} & = & \frac{1}{a_{2}\left(Q_{2}+Ca^{2+}\right)}, \end{array}$$

where

(16) $$\begin{array}{r} Q_{2}=d_{2}\frac{IP_{3}+d_{1}}{IP_{3}+d_{3}}. \end{array}$$

*J*_*chan*_ is given by

(17) $$\begin{array}{r} J_{chan}=r_{C}m_{\infty }^{3}n_{\infty }^{3}h_{\infty }^{3}\left(C_{0}-\left(1+C_{1}\right)Ca^{2+}\right), \end{array}$$

where *r*_*C*_ is the maximal Calcium-Induced Calcium Release (CICR) rate, *C*_0_ is the total free *Ca*^2+^ cytosolic concentration, *C*_1_ is the ER/cytoplasm volume ratio, and *m*_∞_ and *n*_∞_ are the *IP*_3_ Induced Calcium Release (IICR) and CICR channels respectively, which are given by

(18) $$\begin{array}{r} m_{\infty }=\frac{IP_{3}}{IP_{3}+d_{1}}, \end{array}$$

and

(19) $$\begin{array}{r} n_{\infty }=\frac{Ca^{2+}}{Ca^{2+}+d_{5}}. \end{array}$$

*J*_*leak*_ and *J*_*pump*_ are described by

(20) $$\begin{array}{r} J_{leak}=r_{L}\left(C_{0}-\left(1+C_{1}\right)Ca^{2+}\right), \end{array}$$

and

(21) $$\begin{array}{r} J_{pump}=v_{ER}\frac{{\left(Ca^{2+}\right)}^{2}}{k_{ER}^{2}+{\left(Ca^{2+}\right)}^{2}}, \end{array}$$

where *r*_*L*_ is the *Ca*^2+^ leakage rate, *v*_*ER*_ is the maximum SERCA pump uptake rate and *k*_*ER*_ is the SERCA pump activation constant.

The intracellular astrocytic calcium dynamics are used to regulate the release of glutamate from the astrocyte: the Glu pathway. To model this release, it is assumed that when *Ca*^2+^ crosses the CICR threshold, a quantity of glutamate is released (Wade et al., 2012). It is described by

(22) $$\begin{array}{r} \frac{d\left(Glu\right)}{dt}=\frac{-Glu}{\tau_{Glu}}+r_{Glu}\delta \left(t-t_{Ca}\right), \end{array}$$

where *Glu* is the quantity of released glutamate, τ_*Glu*_ is decay rate of glutamate, *r*_*Glu*_ is production rate of glutamate, and *t*_*Ca*_ is the time at which *Ca*^2+^ crosses the threshold. The released glutamate drives the generation of e-SP (Wade et al., 2012). The level of e-SP is modeled by

(23) $$\begin{array}{r} \tau_{eSP}\frac{d\left(eSP\right)}{dt}=-Glu+m_{eSP}Glu\left(t\right), \end{array}$$

where τ_*eSP*_ is the decay rate of Glu, and *m*_*eSP*_ is a constant weight used to control the height of e-SP. It shows that the e-SP level depends on the glutamate released from the astrocyte cell.

The model of DSE in the approach of Wade et al. (2012) is used to describe the relationship between the DSE and the released 2-AG from postsynaptic neuron. The DSE is assumed to change linearly with the cytosolic concentration of 2-AG, which is described by

(24) $$\begin{array}{r} DSE=AG\times K_{AG}, \end{array}$$

where AG is the concentration of 2-AG and *K*_*AG*_ is the scaling factor for the DSE.

### 2.5. Synapse Model

For the synapse, a probabilistic model is employed which is based on the failure and success mechanisms of synaptic neurotransmitter release (Navarrete and Araque, 2010; Wade et al., 2012). A uniformly distributed pseudo-random number generator is used. If the generated random number *rand* is less than or equal to the PR, a current *I*_*inj*_ is injected into the neuron which is shown by

(25) $$I_{syn}^{i}(t)=\{\begin{array}{ll} r_{I}*w_{syn}^{i}(t), & rand\leq PR\\ 0, & rand>PR \end{array}$$

where *r*_*I*_ is the current production rate, and $w_{syn}^{i}$ is the weight of the *i*th synapse. The associated PR of each synapse is determined by the DSE and e-SP together, which is given by

(26) $$\begin{array}{r} PR\left(t\right)=PR\left(t_{0}\right)+DSE\left(t\right)/100+eSP\left(t\right)/100, \end{array}$$

where *PR*(*t*_0_) is the initial PR for each synapse. As discussed in section 2.1, the PR can switch on/off learning at the synaptic terminal by modulating the height of the plasticity learning window. The authors are not aware of any biophysical model that relates PR to the plasticity window weighting parameter *A*_0_ and therefore in this work it is assumed that *A*_0_ is modulated according to

(27) $$A_{0}=\{\begin{array}{ll} 0, & PR\leq PR^{*}\\ (PR-PR^{*})*r, & PR>PR^{*} \end{array}$$

where PR^*^ is the learning activation level and *r* is a constant value which controls the maximum height of the learning window. The STDP rule used in this approach to update the synaptic weights according to the timing difference between the post and presynaptic spikes is described by

(28) $$\delta w(\Delta t)=\{\begin{array}{ll} -A_{0}exp(\frac{\Delta t}{\tau_{+}}), & \Delta t\leq 0\\ A_{0}exp(\frac{-\Delta t}{\tau_{-}}), & \Delta t>0 \end{array}$$

where δ*w*(Δ*t*) is the weight update, Δ*t* is the time difference between the post and presynaptic spikes, *A*_0_ is the height of the plasticity window which limits the maximum levels of weight potentiation and depression, and τ_+_ and τ_−_ control the width of the plasticity window. A symmetrical plasticity window is assumed in this approach, and τ_+_ = τ_−_ = 40*ms*.

From the proposed models, it can be seen that if the *f*_*pre*_ is large enough, *IP*_3_ is generated sufficiently to cause *Ca*^2+^ oscillations. Then the *Ca*^2+^-induced glutamate binds to the mGluRs receptors at the presynaptic terminal resulting in an increase of the synaptic transmission probability PR. Note that the authors wish to point out that astrocytes are believed to gate LTP and LTD by regulating glutamate levels in the synaptic cleft (Foncelle et al., 2018). Since there are many complex biophysical mechanisms involved in the regulation of glutamate, which are still under debate, the authors take the view that modulating the STDP plasticity window using PR is an effective way to capture this gating function. Elevating PR opens the synaptic plasticity learning window and over time *f*_*post*_ gradually increases which, via the 2-AG pathway, contributes to astrocytic *IP*_3_ level until the *Ca*^2+^ oscillation stops. This is accompanied by a reduction in the Glu (e-SP) pathway and PR falls causing a reduction in *f*_*post*_. Therefore, the bursting activity of the postsynaptic neuron is regulated by the GABA interneuron and the astrocyte cell. The results in the next section show the signaling pathways leading to a bursting postsynaptic neuron.

## 3. Results

This section provides simulation results which highlight the dynamic behavior at the synapse terminals and how the interactions between an astrocyte and GABA interneuron can give rise to bursting behavior. The MATLAB simulation platform is used in this work together with the Euler method with the time step of 1 ms. Tables A1, A2 give all the model parameters.

### 3.1. Bursting Output Spike Pattern

**Figure 3:**
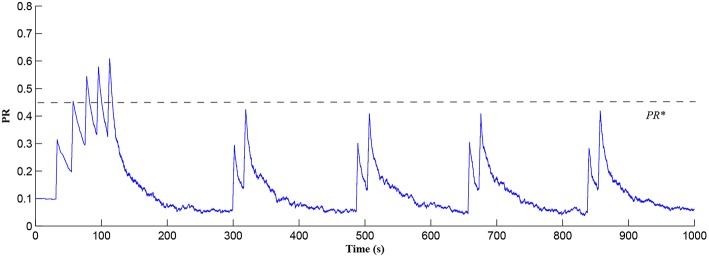
PR as a function of Glu (e-SP) and DSE signals over time. Note that the plasticity window will only be open when *PR*>PR^*^ and this can be observed in Figure 4.

**Figure 4:**
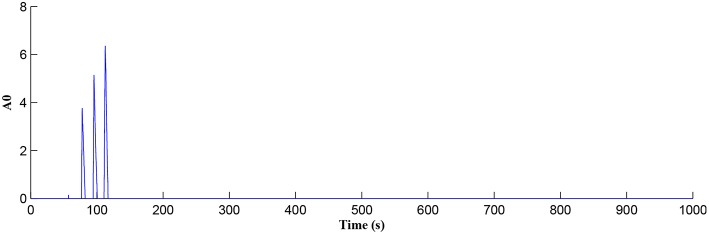
Plasticity window height *A*_0_ as a function of time. Note that PR acts as a switch to open/close the plasticity window controlling the learning period.

**Figure 5:**
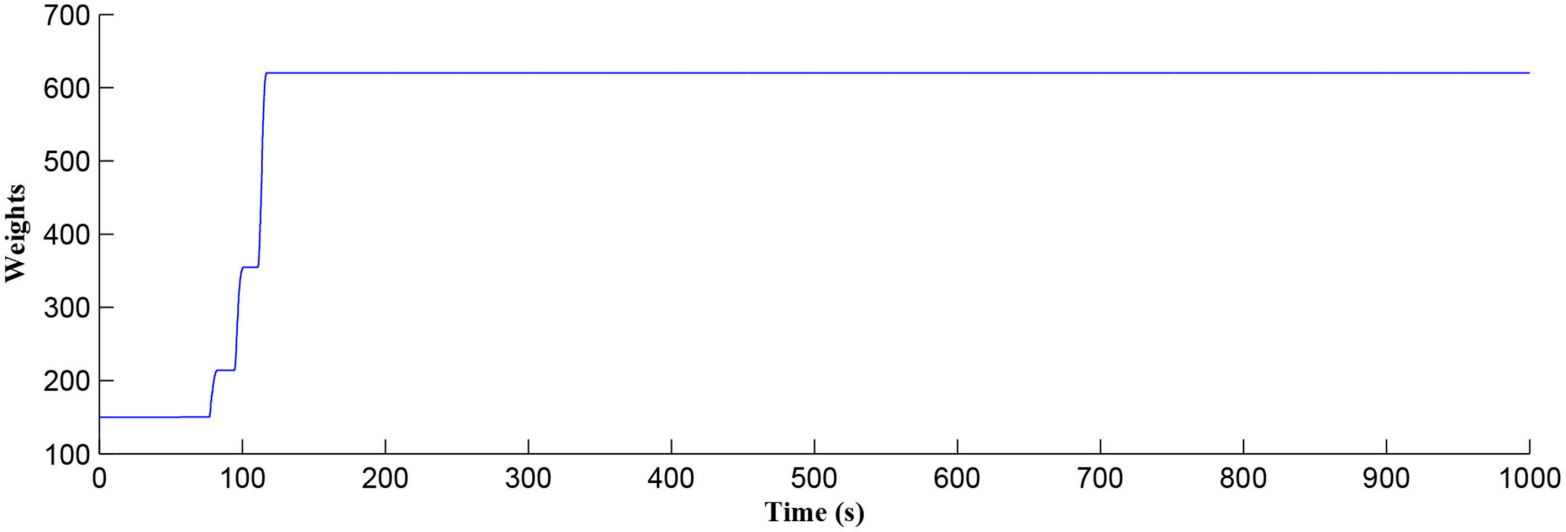
Time dependant synaptic weight update governed by the STDP learning rule. When the plasticity window is open, learning commences, the synaptic weight begins to potentiate and after a period of learning the window shuts off and the synaptic weight stabilizes.

In this simulation both the presynaptic excitatory neuron and the GABA interneuron are stimulated by the same spike train at frequency *f*_*pre*_ = *f*_*GABA*_ which causes the release of GABA and glutamate (Perea et al., 2016). The presynaptic excitatory neuron/GABA interneuron stimulus is 40 Hz in the following simulations as this is sufficient to produce a cytosolic [*IP*_3_]>0.5μ*M*. With *PR*>PR^*^ (see Figure 3), a significant increase occurs in the level of neurotransmitter in the cleft, the learning window opens (Figure 4) and weight potentiation starts (Figure 5) resulting in postsynaptic firing. Note that in Figure 4, the plasticity window height parameter *A*_0_ increases periodically with a corresponding potentiation of the synaptic weight (Figure 5). In our model the resting level for PR is 0.1 and based on the model in Equation (27), if *PR*>PR^*^ (PR^*^ = 0.45 in this work), the STDP learning window opens (*A*_0_>0) at ~80 s, as shown in Figure 4. After the synaptic weight is potentiated, the synapse generates a depolarising current only when the input stimulus is presented at the presynaptic terminal and the PR value is greater than the value of a random number (see probabilistic-based synapse model in Equation 25): this current is injected into the postsynaptic LIF neuron. This injected current increases the postsynaptic potential and the neuron fires a spike if the membrane potential is greater than the firing threshold, *v*_*th*_.

**Figure 6:**
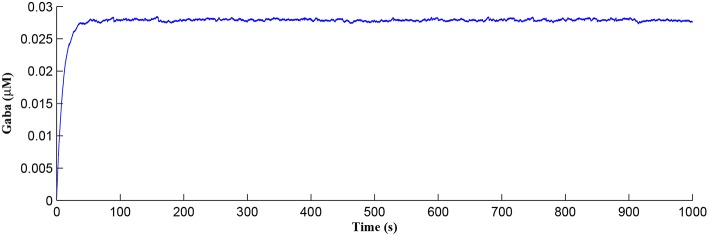
GABA released from the GABA interneuron as a function of time. Under the input spike stimulus of *f*_*GABA*_, GABA increases and then stabilized.

**Figure 7:**
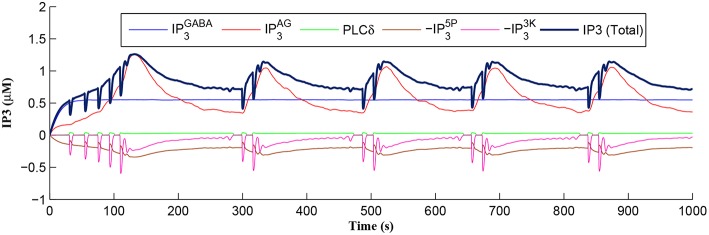
*IP*_3_ dynamics within the astrocyte cell over time. The overall *IP*_3_ includes contributions from $IP_{3}^{GABA}$, $IP_{3}^{AG}$, *PLCδ*, $IP_{3}^{5P}$, and $IP_{3}^{3K}$, where the degradations of *IP*_3_, $IP_{3}^{5P}$, and $IP_{3}^{3K}$, are shown as negative values.

After a period of learning the postsynaptic neuron activity has stabilized and PR drops sufficiently, toward the end of the first set of PR “spikes” (see Figure 3), closing the plasticity window (*A*_0_ = 0) and the weight stabilizes to ~610 at 110 s, as shown in Figure 5: note that because the postsynaptic neuron is now active, *PR*<PR^*^ for all subsequent *Ca*^2+^ oscillations as the DSE pathway is also active. Figure 6 shows the amount of GABA released by the GABA interneuron as a function of time where, as expected, GABA increases gradually and then stabilizes at 0.027 μ*M* under the input spike stimulus. $IP_{3}^{GABA}$ is shown in Figure 7 (blue) as a function of time and stabilizes at ~0.58μ*M* which is consistent with the input stimuli profile. Figure 7 shows the other *IP*_3_ sources that contribute to the total *IP*_3_ in the cytosol.

**Figure 8:**
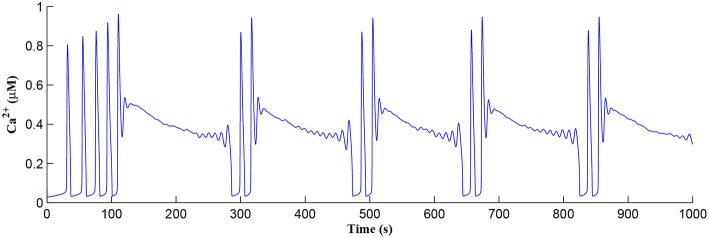
*Ca*^2+^ oscillations in the astrocyte cell as a function of time. Note a longer oscillatory period at the start due to learning but thereafter the oscillator period stabilizes with constant on/off ratio.

Initially the total *IP*_3_ increases with $IP_{3}^{GABA}$ until the *T*_*CICR*_ level is reached triggering the release of *Ca*^2+^, as shown in Figure 8. We have observed from our model that *T*_*CICR*_ is consistent with an *IP*_3_ level of approximately 0.5 μ*M* and whenever *IP*_3_ exceeds this threshold a transient elevation in *Ca*^2+^ occurs, as can be seen in Figure 8. Note however that as the *IP*_3_ level increases with $IP_{3}^{AG}$ the degradation in *IP*_3_ due to elevated *Ca*^2+^/*IP*_3_ levels is insufficient to reduce *IP*_3_ to below 0.5 μ*M* and consequently the transient elevations of *Ca*^2+^ stops just after 100 s followed by a relatively slow degradation of *IP*_3_ and *Ca*^2+^: these periodic bursts in *Ca*^2+^ gives rise to a *Ca*^2+^ oscillatory wave where the initial *Ca*^2+^ burst is longer due to synaptic potentiation. At the onset of each subsequent *Ca*^2+^ burst the *IP*_3_ level drops sharply and we attribute this to strong dependence of $IP_{3}^{3K}$ on *Ca*^2+^ (Equation 10). As the *Ca*^2+^ level drops *J*_*chan*_ (Equation 17) reverses direction perturbing the rate of change in *Ca*^2+^ (Equation 12) and this causes a rapid increase in $IP_{3}^{3K}$ and a corresponding decrease in *IP*_3_ (Equation 11).

**Figure 9:**
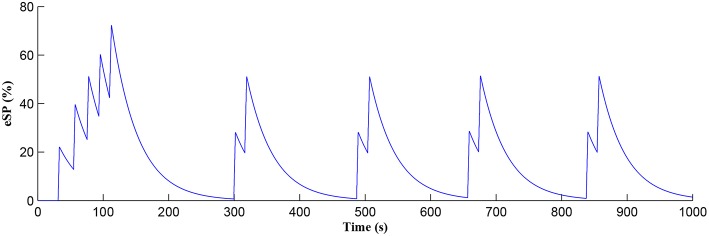
Glu (e-SP) signaling pathway as a function of time. Note a longer period of elevation of Glu (e-SP) at the start due to synaptic potentiation but thereafter the Glu (e-SP) profile repeats in time.

**Figure 10:**
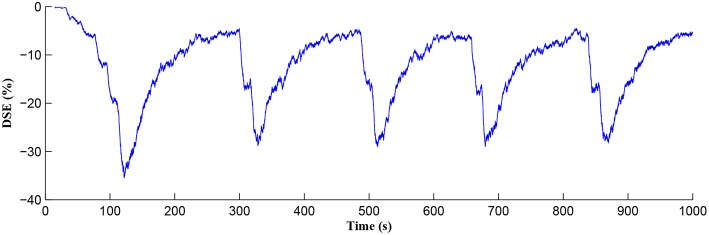
DSE signaling pathway from the postsynaptic neuron, which correlates with the activity of the postsynaptic neuron.

The *Ca*^2+^ oscillation is initiated at ~20 s (*T*_*CICR*_ is exceeded; Figure 8) and this triggers the release of glutamate targeting group I mGluRs on the presynaptic terminal, i.e., Glu (e-SP) pathway is activated (Figure 9). Figure 9 shows that the Glu (e-SP) signal accumulates at each CICR and rapidly decays after the *Ca*^2+^ transients have ceased at ~120 s. Also the DSE pathway increases as the activity of the postsynaptic neuron is increasing, and competes with the Glu (e-SP) pathway to restrict PR to a relatively stable low value for all subsequent *Ca*^2+^ oscillations that occur post-learning, as shown in Figure 10. Again note a longer period of elevation of the DSE signal at the start due to synaptic potentiation but thereafter the DSE profile repeats in time.

**Figure 11:**
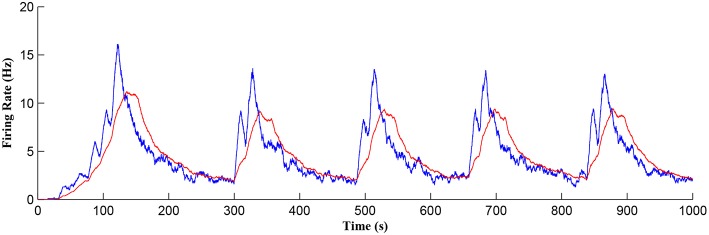
Firing rate of the postsynaptic neuron where a continual bursting behavior is evident. The firing activity was calculated using a sliding time window of 10 s (blue) and again for a sliding window of 40 s (red) where the latter gives a better average.

Figure 11 shows the firing rate of the postsynaptic neuron, which is calculated based on a sliding time window of 10 s (blue) and 40 s (red), respectively. Note that the first burst reaches a higher level of postsynaptic neuron activity when compared to subsequent bursts. Also, between bursts the activity never falls back to zero. This is because the first burst occurs during the weight potentiation phase when the synaptic weight is continually updated and eventually stabilized, whereas in all subsequent neuronal bursts no weight potentiation occurs. Clearly from Figure 11 a continual postsynaptic bursting behavior is evident.

**Figure 12:**
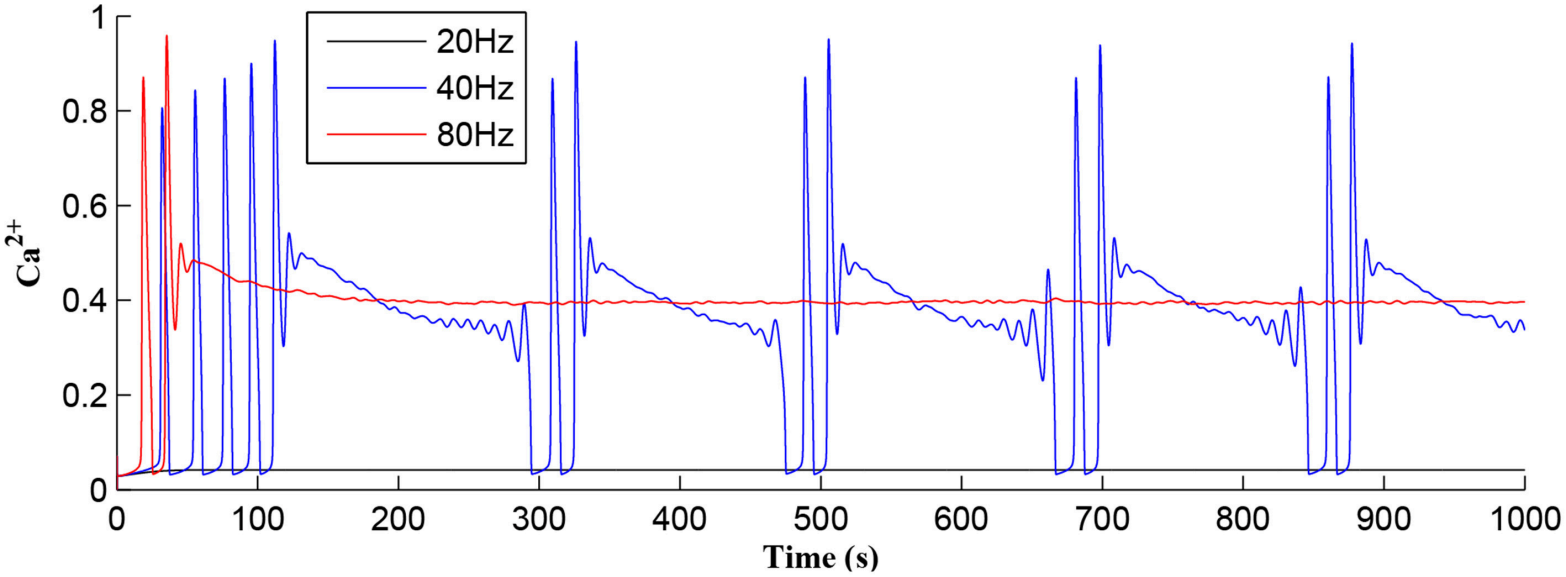
Astrocytic *Ca*^2+^ as a function of time with *f*_*pre*_ of 20, 40, and 80 Hz as a parameter. Note that for the extreme cases of (20 or 80 Hz) no *Ca*^2+^ oscillations occur: for *f*_*pre*_ = 20 Hz *T*_*CICR*_ can never be achieved and at 80Hz degradation of *IP*_3_ is insufficient to allow CICR to repeatedly occur. Consequently, there is a frequency window over which oscillations can occur.

Referring to Figure 12, we show simulations for *f*_*pre*_ of 20, 40, and 80 Hz where clearly only *f*_*pre*_ = 40*Hz* results in repeated *Ca*^2+^ oscillations. This is because at 20Hz the *T*_*CICR*_ level cannot be reached whereas at 80Hz the astrocyte cytosol is quickly swamped with both *IP*_3_ and *Ca*^2+^ and subsequent degradation in *IP*_3_ is insufficient to allow further CICR. Consequently our model shows presynaptic frequency selectivity which is consistent with work reported elsewhere (Bienenstock et al., 1982; Dong et al., 2015).

In addition, as the morphology of GABA interneurons and receptor density at the astrocyte cell differ, the $IP_{3}^{GABA}$ levels vary under the same input *f*_*pre*_. $IP_{3}^{GABA}$ is a main contributor to the total *IP*_3_, thus the greater $IP_{3}^{GABA}$, the longer the process of *IP*_3_ degradation.

**Figure 13:**
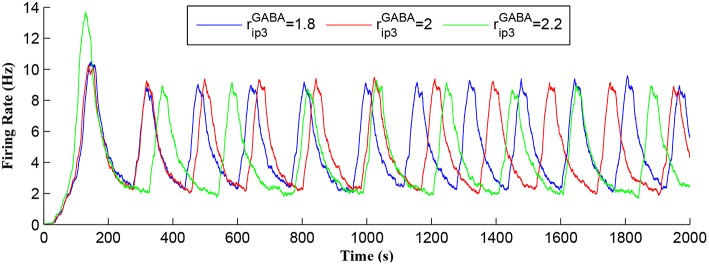
Postsynaptic neuron burst firing as a function of time with $IP_{3}^{GABA}$ production rates ($r_{ip3}^{GABA}$) as a parameter. When $r_{ip3}^{GABA}$ increases, $IP_{3}^{GABA}$ level increases and the frequency of the bursting decreases. Due to a high $IP_{3}^{GABA}$ level, a long time period is required to degrade the overall *IP*_3_ and to restart the *Ca*^2+^ oscillation. Thus, the bursting frequency is low.

Figure 7 shows that when *IP*_3_ degrades sufficiently to once again enable *IP*_3_ to cross *T*_*CICR*_ from below, a transient elevation in *Ca*^2+^ results and PR increases with a corresponding increase in the postsynaptic neuron burst frequency. Therefore, different $IP_{3}^{GABA}$ levels lead to different burst frequencies of the postsynaptic neuron. To determine the dependency of neuronal burst frequency on the production rate of $IP_{3}^{GABA}$, $r_{ip3}^{GABA}$, a simulation was carried out (Figure 13) which shows the firing rates of the postsynaptic neuron under different production rates with *f*_*pre*_ fixed at 40 Hz. It can be seen that when the $r_{ip3}^{GABA}$ increases, the frequency of the bursting decreases. For example, for the first 1,000 s, there are 6, 5, 4 bursts under the $IP_{3}^{GABA}$ production rates ($r_{ip3}^{GABA}$) of 1.8, 2, and 2.2, respectively. This is because a high $IP_{3}^{GABA}$ level requires a significant time period to degrade the total *IP*_3_, and to restart the *Ca*^2+^ oscillation and bursting behavior, thus the bursting frequency is low. Note that a fixed frequency of the input stimulus (i.e., *f*_*pre*_ = 40 Hz) is used in this experiment, however the same results are observed for other *f*_*pre*_ values such as 50 Hz, and the burst frequency variation is not constrained for specific *f*_*pre*_ values. The results in Figures 12, 13 demonstrate the functionalities of the GABA interneuron including the presynaptic frequency selectivity and postsynaptic bursting frequency regulation.

## 4. Conclusions

In this paper, a biophysical model is proposed where it is shown that GABA interneuron regulates the astrocytic *IP*_3_ secondary messenger and thus the probability of release (PR) at the presynaptic terminal. In our model we propose that PR modulates the height of the plasticity window and therefore controls when synaptic potentiation/depression occurs. Specifically, the simulations show that during the weight potentiation phase, increasing *IP*_3_ leads to a cycle of CICR events where each is followed by rapid degradation in *IP*_3_. Over time the firing frequency of the postsynaptic neuron continually increases and eventually the synaptic weights stabilize. Postsynaptic firing results in the release of 2-AG into the extracellular space and this messenger binds to CB1R receptors on the astrocyte membrane. The associated $IP_{3}^{AG}$ contributes to the total cytosolic *IP*_3_ and eventually *Ca*^2+^ oscillations, and therefore the Glu (e-SP) pathway ceases: 2-AG also binds to CB1Rs on the presynaptic terminal causing a decrease of the synaptic transmission PR via the DSE pathway. PR therefore decreases at the presynaptic terminal which reduces the level of neurotransmitter in the cleft, and consequently the firing frequency of the postsynaptic neuron diminishes, as does $IP_{3}^{AG}$. Thereafter, the total *IP*_3_/*Ca*^2+^ degrades significantly over time but is replenished by $IP_{3}^{GABA}$ and a subsequent cycle of CICR events commences—the Glu (e-SP) pathway is re-established with an associated increase in PR and the level of neurotransmitter in the cleft is raised. However, in this instance weight potentiation does not occur as *PR*<PR^*^. The postsynaptic neuron firing rate increases again until the *Ca*^2+^ transients stop and thereafter the activity of the postsynaptic neuron falls off again. A network burst has occurred and this is followed by repeated bursts where each coincides with *Ca*^2+^ transients: the network burst frequency correlates with the *Ca*^2+^ oscillatory wave. In addition, the GABA released by the GABA interneuron controls the frequency range within which the network bursts can occur. Future work will further explore other neurotransmitters released by astrocytes such as D-serine and ATP, and also slow inward currents at the postsynaptic terminal as a result of glutamate release by astrocytes.

The authors recognize that this study is based on biological findings of the simplest signaling mechanisms involving astrocytic GABA responses and astrocytic glutamate signaling in presynaptic terminals that regulate network function. Other factors, such as astrocytic ATP/adenosine release from astrocytes (Covelo and Araque, 2018), are not considered in the present model, but may also contribute to further shape of network activity, adding further complexity of the network effects of astrocyte signaling. Further studies incorporating these additional elements are therefore required to get a complete view of the astrocyte roles in network function. Despite this the present findings have potential implications for the generation of normal and pathologic circuit behavior in the brain, relevant to brain diseases that feature altered synaptic properties or where there is a propensity for the episodic synchronized bursting behavior of neurons. The electroencephalogram (EEG) is a composite product of population-level neuronal firing patterns of differing frequencies. Our findings suggest that GABA-B signaling via astrocytes may be relevant to the generation of certain frequencies and behaviors in the EEG. Seizures are the hallmark of the common brain disease epilepsy and are generated by hyper-synchronous discharges of populations of neurons. Notably, gene expression levels of key components modeled here, including the *IP*_3_ receptor and GABA-B receptor, are dysregulated in human epileptic brain tissue or animal models (Matsumoto et al., 1996; Nishimura et al., 2005; Sheilabi et al., 2018) of epilepsy. Mutations in these genes have also been identified in individuals with epilepsy (Møller et al., 2017; Yoo et al., 2017). Indeed, the GABA-B receptor is a long-standing therapeutic target for the treatment of epilepsy (Bowery, 2006), and more recently the *IP*_3_ receptor was reported to be a target of levetiracetam, one of the most effective anti-epileptic drugs (Nagarkatti et al., 2008). The present model offers a novel mechanism to explain how astrocyte-neuron interactions regulate seizure-like activity (Gómez-Gonzalo et al., 2010), and how alterations in the described pathways may contribute to hyper-synchronous firing. It may also offer therapeutic insights through targeted manipulation of the astrocytic GABA-B or *IP*_3_ systems followed by evaluation of the resting electroencephalogram (EEG) and investigating whether this alters the frequency or occurrence of pathophysiological neuronal firing and seizures.

## Data Availability

The raw data supporting the conclusions of this manuscript will be made available by the authors, without undue reservation, to any qualified researcher.

## Author Contributions

JL, LM, AA, JW, JHa, SK, DCH, NC, AT, JT, and DMH investigated and proposed the biophysical model. JL, LM, AA, JW, JHa, DCH, NC, and DMH wrote and revised the manuscript. JL, LM, AA, JW, JHa, DCH, NC, AJ, AT, JT, AM, JHi, and DMH analyzed the results and reviewed the manuscript.

### Conflict of Interest Statement

The authors declare that the research was conducted in the absence of any commercial or financial relationships that could be construed as a potential conflict of interest.

## Appendix

**Table A1:** GABA interneuron, neuron, and synapse parameters.

**Parameter**	**Parameter description**	**Value**	**Source**
τ_*GABA*_	GABA decay rate	10 s	-
*r*_*GABA*_	GABA production rate	0.07 μ*Ms*^−1^	-
τ_*m*_	Neuron membrane time constant	24 ms	-
*R*_*m*_	Neuron membrane resistance	1.2GΩ	Wade et al., 2012
τ_*AG*_	2-AG decay rate	10 s	Wade et al., 2012
*r*_*AG*_	2-AG production rate	0.27 μ*Ms*^−1^	Wade et al., 2012
*r*_*I*_	Synaptic current production rate	16	-
*PR*^*^	Learning activation level	0.45	-
*r*	Maximum height weighting factor of learning window	40	-
τ_+_	Potentiation width of the plasticity window	40 ms	Liu et al., 2019
τ_−_	Depression width of the plasticity window	40 ms	Liu et al., 2019

**Table A2:** Astrocyte cell parameters.

**Parameter**	**Parameter description**	**Value**	**Source**
$IP_{3}^{GABA*}$	Baseline value of $IP_{3}^{GABA}$	0.16 μ*M*	-
$\tau_{ip3}^{GABA}$	Decay rate of $IP_{3}^{GABA}$	7 s	-
$r_{ip3}^{GABA}$	Production rate of $IP_{3}^{GABA}$	2 μ*M*	-
$IP_{3}^{AG*}$	Baseline value of $IP_{3}^{AG}$	0.16 μ*M*	Wade et al., 2012
$\tau_{ip3}^{AG}$	Decay rate of $IP_{3}^{AG}$	7 s	Wade et al., 2012
$r_{ip3}^{AG}$	Production rate of $IP_{3}^{AG}$	5 μ*M*	Wade et al., 2012
*K*_*PLCδ*_	*Ca*^2+^ affinity of PLCδ	0.1 μM	De Pittà et al., 2009
*K*_δ_	Inhibition constant of PLCδ activity	1.5 μM	De Pittà et al., 2009
${\bar{r}}_{5P}$	*IP*_3_ degradation rate by IP-5P	0.27	-
${\bar{v}}_{3K}$	Maximum degradation rate by *IP*_3_-3K	2	De Pittà et al., 2009
*K*_*D*_	*Ca*^2+^ affinity of *IP*_3_-3K	0.7	De Pittà et al., 2009
*K*_3_	*IP*_3_ affinity of *IP*_3_-3K	1	De Pittà et al., 2009
*r*_*C*_	Maximal CICR rate	6 *s*^−1^	De Pittà et al., 2009
*r*_*L*_	*Ca*^2+^ leakage rate from ER	0.11 *s*^−1^	De Pittà et al., 2009
*v*_*ER*_	Maximum SERCA pump uptake rate	0.9 μ*Ms*^−1^	De Pittà et al., 2009
*k*_*ER*_	SERCA pump activation constant	0.1 μ*M*	De Pittà et al., 2009
*r*_*Glu*_	Production rate of glutamate	65 μ*Ms*^−1^	-
τ_*Glu*_	Decay rate of glutamate	0.1s	Wade et al., 2012
*m*_*eSP*_	e-SP weighting factor	35000	-
τ_*eSP*_	Decay rate of e-SP	40 s	Wade et al., 2012
*a*_2_	IP3R *Ca*^2+^ inactivation binding rate	0.2 μ*Ms*^−1^	Wade et al., 2012
*c*_0_	Total free *Ca*^2+^ cytosol concentration	2 μ*M*	Wade et al., 2012
*c*_1_	Ratio of ER volume to cytosol volume	0.185	Wade et al., 2012
*d*_1_	*IP*_3_ dissociation constant	0.13 μ*M*	Wade et al., 2012
*d*_2_	*Ca*^2+^ inactivation dissociation constant	1.049 μ*M*	Wade et al., 2012
*d*_3_	*IP*_3_ dissociation constant	0.9434 μ*M*	Wade et al., 2012
*d*_5_	*Ca*^2+^ activation dissociation constant	0.08234 μ*M*	Wade et al., 2012
*Ca*^2+^ threshold	Astrocyte glutamate release *Ca*^2+^ threshold	0.7 μ*M*	-
*K*_*AG*_	DSE weighting factor	1,000	-

## References

[B1] Alger B. E. (2002). Retrograde signaling in the regulation of synaptic transmission: focus on endocannabinoids. Progr. Neurobiol. 68, 247–286. 10.1016/S0301-0082(02)00080-1 12498988

[B2] Araque A. Parpura V. Sanzgiri R. P. Haydon P. G. (1999). Tripartite synapses: glia, the unacknowledged partner. Trends Neurosci. 22, 208–215. 10.1016/S0166-2236(98)01349-6 10322493

[B3] Arichi T. Whitehead K. Barone G. Pressler R. Padormo F. Edwards A. D.. (2017). Localization of spontaneous bursting neuronal activity in the preterm human brain with simultaneous EEG-fMRI. eLife 6:27814. 10.7554/eLife.27814 28893378 PMC5595428

[B4] Bienenstock E. L. Cooper L. N. Munro P. W. (1982). Theory for the development of neuron selectivity: orientation specificity and binocular interaction in visual cortex. J. Neurosci. 2, 32–48. 10.1523/JNEUROSCI.02-01-00032.1982 7054394 PMC6564292

[B5] Bowery N. G. (2006). GABAB receptor: a site of therapeutic benefit. Curr. Opin. Pharmacol. 6, 7–43. 10.1016/j.coph.2005.10.002 16361115

[B6] Breslin K. Wade J. J. Wong-Lin K. Harkin J. Flanagan B. Van Zalinge H.. (2018). Potassium and sodium microdomains in thin astroglial processes: a computational model study. PLoS Comput. Biol. 14:e1006151. 10.1371/journal.pcbi.1006151 29775457 PMC5979043

[B7] Covelo A. Araque A. (2018). Neuronal activity determines distinct gliotransmitter release from a single astrocyte. eLife 7:32237. 10.7554/eLife.32237 29380725 PMC5790377

[B8] Dawson A. P. (1997). Calcium signalling: how do IP3 receptors work? Curr. Biol. 7, 544–547. 10.1016/S0960-9822(06)00277-6 9285705

[B9] De Pittà M. Goldberg M. Volman V. Berry H. Ben-Jacob E. (2009). Glutamate regulation of calcium and IP3 oscillating and pulsating dynamics in astrocytes. J. Biol. Phys. 35, 383–411. 10.1007/s10867-009-9155-y 19669422 PMC2750743

[B10] Dong W. S. Zeng F. Lu S. H. Liu A. Li X. J. Pan F. (2015). Frequency-dependent learning achieved using semiconducting polymer/electrolyte composite cells. Nanoscale 7, 16880–16889. 10.1039/C5NR02891D 26412715

[B11] Flanagan B. McDaid L. Wade J. Wong-Lin K. Harkin J. (2018). A computational study of astrocytic glutamate influence on post-synaptic neuronal excitability. PLoS Comput. Biol. 14, 1–25. 10.1371/journal.pcbi.1006040 29659572 PMC5919689

[B12] Foncelle A. Mendes A. Jedrzejewska-Szmek J. Valtcheva S. Berry H. Blackwell K. T.. (2018). Modulation of spike-timing dependent plasticity : towards the inclusion of a third factor in computational models. Front. Comput. Neurosci. 12, 1–21. 10.3389/fncom.2018.00049 30018546 PMC6037788

[B13] Gabbiani F. Metzner W. Wessel R. Koch C. (1996). From stimulus encoding to feature extraction in weakly electric fish. Nature 38, 564–567. 10.1038/384564a0 8955269

[B14] Gerstner W. Kistler W. M. (2002). Spiking Neuron Models: Single Neurons, Populations, Plasticity. Cambridge University Press.

[B15] Ghosh-dastidar S. Adeli H. (2009). Spiking neural networks. Int. J. Neural Syst. 19, 295–308. 10.1142/S0129065709002002 19731402

[B16] Gómez-Gonzalo M. Losi G. Chiavegato A. Zonta M. Cammarota M. Brondi M.. (2010). An excitatory loop with astrocytes contributes to drive neurons to seizure threshold. PLoS Biol. 8:e1000352. 10.1371/journal.pbio.1000352 20405049 PMC2854117

[B17] Halassa M. M. Fellin T. Takano H. Dong J. Haydon P. G. (2007). Synaptic islands defined by the territory of a single astrocyte. J. Neurosci. 27, 6473–6477. 10.1523/JNEUROSCI.1419-07.2007 17567808 PMC6672436

[B18] Hu J. Tang H. Tan K. C. Li H. Shi L. (2013). A spike-timing-based integrated model for pattern recognition. Neural Comput. 25, 450–472. 10.1162/NECO_a_00395 23148414

[B19] Izhikevich E. M. (2003). Simple model of spiking neurons. IEEE Trans. Neural Netw. 14, 1569–1572. 10.1109/TNN.2003.820440 18244602

[B20] Johnson A. P. Liu J. Millard A. G. Karim S. Tyrrell A. M. Harkin J. (2018). Homeostatic fault tolerance in spiking neural networks: a dynamic hardware perspective. IEEE Trans. Circ. Syst. 65, 687–699. 10.1109/TCSI.2017.2726763

[B21] Kullmann D. M. (2011). Interneuron networks in the hippocampus. Curr. Opin. Neurobiol. 21, 709–716. 10.1016/j.conb.2011.05.006 21636266

[B22] Kurosinski P. Götz J. (2002). Glial cells under physiologic and pathologic conditions. Arch. Neurol. 59, 1524–1528. 10.1001/archneur.59.10.1524 12374489

[B23] Li Y.-X. Rinzel J. (1994). Equations for InsP3 receptor-mediated calcium oscillations derived from a detailed kinetic model: a Hodgkin-Huxley like formalism. J. Theor. Biol. 166, 461–473. 10.1006/jtbi.1994.1041 8176949

[B24] Liu J. Harkin J. Maguire L. P. Mcdaid L. J. Wade J. J. (2018). SPANNER: a self-repairing spiking neural network hardware architecture. IEEE Trans. Neural Netw. Learn. Syst. 29, 1287–1300. 10.1109/TNNLS.2017.2673021 28287992

[B25] Liu J. Harkin J. McElholm M. McDaid L. Jimenez-Fernandez A. Linares-Barranco A. (2015). “Case study: bio-inspired self-adaptive strategy for spike-based PID controller,” in IEEE International Symposium on Circuits and Systems (ISCAS) (Lisbon), 2700–2703.

[B26] Liu J. McDaid L. J. Harkin J. Karim S. Johnson A. P. Millard A. G.. (2019). Exploring self-repair in a coupled spiking astrocyte neural network. IEEE Trans. Neural Netw. Learn. Syst. 30, 865–875. 10.1109/TNNLS.2018.2854291 30072349

[B27] Lüscher C. Malenka R. C. (2012). NMDA receptor-dependent long-term potentiation and long-term depression (LTP/LTD). Cold Spring Harb. Perspect. Biol. 4, 1–15. 10.1101/cshperspect.a005710 22510460 PMC3367554

[B28] Magee J. C. Johnston D. (1997). A synaptically controlled, associative signal for synaptic plasticity in hippocampal neurons. Science 275, 209–213. 10.1126/science.275.5297.209 8985013

[B29] Marchant J. Callamaras N. Parker I. (1999). Initiation of IP3-mediated Ca2+ waves in Xenopus oocytes. EMBO J. 18, 5285–5299. 10.1093/emboj/18.19.5285 10508162 PMC1171599

[B30] Matsumoto M. Nakagawa T. Inoue T. Nagata E. Tanaka K. Takano H.. (1996). Ataxia and epileptic seizures in mice lacking type 1 inositol 1,4,5-trisphosphate receptor. Nature 379, 168–171. 10.1038/379168a0 8538767

[B31] Miles R. Wong R. K. (1986). Excitatory synaptic interactions between CA3 neurones in the guinea pig hippocampus. J. Physiol. 373, 397–418. 10.1113/jphysiol.1986.sp016055 3018233 PMC1182545

[B32] Møller R. S. Wuttke T. V. Helbig I. Marini C. Johannesen K. M. Brilstra E. H.. (2017). Mutations in GABRB3 From febrile seizures to epileptic encephalopathies. Neurology 88, 483–492. 10.1212/WNL.0000000000003565 28053010 PMC5278942

[B33] Naeem M. McDaid L. J. Harkin J. Wade J. J. Marsland J. (2015). On the role of astroglial syncytia in self-repairing spiking neural networks. IEEE Trans. Neural Netw. Learn. Syst. 26, 2370–2380. 10.1109/TNNLS.2014.2382334 25576582

[B34] Nagarkatti N. Deshpande L. S. DeLorenzo R. J. (2008). Levetiracetam inhibits both ryanodine and IP3 receptor activated calcium induced calcium release in hippocampal neurons in culture. Neurosci. Lett. 436, 289–293. 10.1016/j.neulet.2008.02.076 18406528 PMC2847576

[B35] Navarrete M. Araque A. (2010). Endocannabinoids potentiate synaptic transmission through stimulation of astrocytes. Neuron 68, 113–126. 10.1016/j.neuron.2010.08.043 20920795

[B36] Nishimura T. Schwarzer C. Gasser E. Kato N. Vezzani A. Sperk G. (2005). Altered expression of GABA-A and GABA-B receptor subunit mRNAs in the hippocampus after kindling and electrically induced status epilepticus. Neuroscience 134, 691–704. 10.1016/j.neuroscience.2005.04.013 15951123

[B37] Perea G. Gómez R. Mederos S. Covelo A. Ballesteros J. J. Schlosser L.. (2016). Activity-dependent switch of GABAergic inhibition into glutamatergic excitation in astrocyte-neuron networks. eLife 5, 1–26. 10.7554/eLife.20362 28012274 PMC5231406

[B38] Reid D. Hussain A. J. Tawfik H. (2014). Financial time series prediction using spiking neural networks. PLoS ONE 9:e103656. 10.1371/journal.pone.0103656 25170618 PMC4149346

[B39] Serrano A. Haddjeri N. Lacaille J.-c. Robitaille R. (2006). GABAergic network activation of glial cells underlies hippocampal heterosynaptic depression. J. Neurosci. 26, 5370–5382. 10.1523/JNEUROSCI.5255-05.2006 16707789 PMC6675310

[B40] Sheilabi M. A. Battacharyya D. Caetano L. Thom M. Reuber M. Duncan J. S.. (2018). Quantitative expression and localization of GABA-B receptor protein subunits in hippocampi from patients with refractory temporal lobe epilepsy. Neuropharmacology 136, 117–128. 10.1016/j.neuropharm.2017.08.001 28782512

[B41] Song S. Miller K. D. Abbott L. F. (2000). Competitive Hebbian learning through spike-timing-dependent synaptic plasticity. Nat. Neurosci. 3, 919–926. 10.1038/78829 10966623

[B42] Wade J. McDaid L. Harkin J. Crunelli V. Kelso S. (2012). Self-repair in a bidirectionally coupled astrocyte-neuron (AN) system based on retrograde signaling. Front. Comput. Neurosci. 6:76. 10.3389/fncom.2012.00076 23055965 PMC3458420

[B43] Wade J. J. McDaid L. J. Harkin J. Crunelli V. Kelso J. A. S. Beiu V. (2011). “Exploring retrograde signaling via astrocytes as a mechanism for self repair,” in International Joint Conference on Neural Networks (IJCNN) (San Jose, CA: IEEE), 3149–3155.

[B44] Yoo Y. Jung J. Lee Y. N. Lee Y. Cho H. Na E.. (2017). GABBR2 mutations determine phenotype in rett syndrome and epileptic encephalopathy. Ann. Neurol. 82, 466–478. 10.1002/ana.25032 28856709

